# The combination of PD-L1 expression and decreased tumor-infiltrating lymphocytes is associated with a poor prognosis in triple-negative breast cancer

**DOI:** 10.18632/oncotarget.14698

**Published:** 2017-01-17

**Authors:** Hitomi Mori, Makoto Kubo, Rin Yamaguchi, Reiki Nishimura, Tomofumi Osako, Nobuyuki Arima, Yasuhiro Okumura, Masayuki Okido, Mai Yamada, Masaya Kai, Junji Kishimoto, Yoshinao Oda, Masafumi Nakamura

**Affiliations:** ^1^ Department of Surgery and Oncology, Graduate School of Medical Sciences, Kyushu University, Fukuoka, Japan; ^2^ Department of Pathology, Kurume University Medical Center, Kurume, Japan; ^3^ Breast Center, Kumamoto Shinto General Hospital, Kumamoto, Japan; ^4^ Department of Pathology, Kumamoto Shinto General Hospital, Kumamoto, Japan; ^5^ Department of Breast and Endocrine Surgery, Kumamoto City Hospital, Kumamoto, Japan; ^6^ Department of Surgery, Hamanomachi Hospital, Fukuoka, Japan; ^7^ Department of Research and Development of Next Generation Medicine, Faculty of Medical Sciences, Kyushu University, Fukuoka, Japan; ^8^ Department of Anatomic Pathology, Graduate School of Medical Sciences, Kyushu University, Fukuoka, Japan

**Keywords:** programmed cell death ligand-1, tumor-infiltrating lymphocytes, triple-negative breast cancer, biomarker, prognosis

## Abstract

This study included patients with primary triple-negative breast cancer (TNBC) who underwent resection without neoadjuvant chemotherapy between January 2004 and December 2014. Among the 248 TNBCs studied, programmed cell death ligand-1 (PD-L1) expression was detected in 103 (41.5%) tumors, and high levels of tumor-infiltrating lymphocytes (TILs) were present in 118 (47.6%) tumors. PD-L1 expression correlated with high levels of TILs, but was not a prognostic factor. Patients with TILs-high tumors had better overall survival than those with TILs-low tumors (*P* = 0.016). There was a strong interaction between PD-L1 expression and TILs that was associated with both recurrence-free survival (*P* = 0.0018) and overall survival (*P* = 0.015). Multivariate Cox proportional hazards model analysis showed that PD-L1-positive/TILs-low was an independent negative prognostic factor for both recurrence-free survival and overall survival. Our findings suggest that PD-L1-positive/TILs-low tumors are associated with a poor prognosis in patients with TNBC, and that it is important to focus on the combination of PD-L1 expression on tumor cells and TILs present in the tumor microenvironment. These biomarkers may be useful for stratification of TNBCs and for predicting prognosis and developing novel cancer immunotherapies.

## INTRODUCTION

Triple-negative breast cancer (TNBC) is characterized by a lack of expression of estrogen receptor (ER), progesterone receptor (PR), and human epidermal growth factor receptor 2 (HER2) and represents up to 20% of all breast cancers. This subtype is a heterogeneous tumor that encompasses other breast cancer molecular subtypes. In general, TNBC is a high-grade, aggressive disease with a high rate of distant metastasis, and is associated with a poorer outcome than other breast cancer subtypes, despite a good response to standard chemotherapy regimens [1]. Therefore, further definition of these subclasses and novel therapeutic strategies are needed to predict prognosis and choose appropriate treatments for patients with TNBC.

Tumor-infiltrating lymphocytes (TILs) have been shown to have prognostic and predictive value in both adjuvant [2–4] and neoadjuvant settings [5–7] in breast cancer, especially in TN and HER2 breast cancers. TILs (both stromal and intratumoral) are associated with high histologic grade, hormone receptor negativity and high Ki-67 expression [2], possibly as a result of the load of somatic mutation. In TNBC in particular, a high number of stromal TILs is predictive of a more favorable outcome, and the prognostic value of stromal TILs can be considered strong evidence. However, according to the International TILs Working Group, TILs should not yet be used as a biomarker for withholding chemotherapy [8].

The programmed cell death protein 1 (PD-1, also known as CD279) pathway plays a crucial role in regulating immune responses. Programmed cell death ligand-1 (PD-L1, ligand for PD-1; also known as B7-H1 or CD274) on tumor cells is upregulated by constitutive oncogenic signaling (innate resistance) or by inflammatory signals in the tumor microenvironment (adaptive resistance), such as interferon-γ (INF-γ) produced by some activated T cells and natural killer cells [9]. Although some results remain controversial [10], PD-L1 expression reportedly correlates with a poor clinical outcome in several types of malignancy, and may be a predictive marker of PD-1/PD-L1 pathway inhibition [11–13]. In TNBC, the value of PD-L1 expression as a biomarker has so far been controversial [14–18] and the underlying molecular mechanisms remain unclear. Therefore, further studies are needed to identify immune biomarkers for the selection of patients who would most likely benefit from novel immunotherapies.

In the present study, we retrospectively analyzed PD-L1 expression and stromal TILs in 248 TNBCs. We also explored the correlation between immunologic features on tumors and immune cells and the clinicopathological characteristics of the tumors, their response to chemotherapy and clinical outcome.

## RESULTS

### Clinicopathological features, PD-L1 expression, and TILs

**Figure 1:**
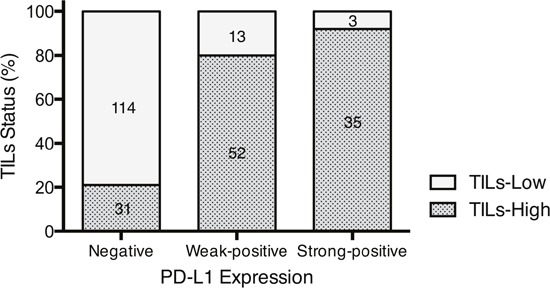
Relationship between PD-L1 expression and TILs status Figures within this bar graph depict absolute numbers of cases. The result of Cochran-Armitage test for trend was *P* < 0.0001.

We evaluated 248 TN tumors with respect to the clinicopathological data (Table 1), PD-L1 expression on tumor cells (Supplementary Table 1 and Supplementary Figure 1) and stromal TILs (Supplementary Figure 2). Among the 248 TN tumors, PD-L1 expression was classified as strong-positive in 38 (15.3%), weak-positive in 65 (26.2%), and negative in 145 (58.5%) (Supplementary Table 1). Stromal TILs were present at a high level in 118 (47.6%) of the tumors (Supplementary Table 2). The breakdown of these results is as follows: high levels of TILs were present in 35 (92.1%) PD-L1 strong-positive tumors, 52 (80.0%) PD-L1 weak-positive tumors, and 31 (21.4%) PD-L1-negative tumors (Figure 1). Positive PD-L1 expression was significantly correlated with high levels of TILs (*P* < 0.0001, Figure 1). Patients with PD-L1-positive tumors were younger than those with PD-L1-negative tumors (*P* = 0.007). The nuclear grade and Ki-67 index were higher in PD-L1-positive tumors than in PD-L1-negative tumors (*P* = 0.0015 and *P* < 0.0001, respectively), although there was no significant difference between the two groups with respect to tumor size, nodal status and pathological stage (Table 1). We also evaluated PD-L1 expression on tumor-infiltrating immune cells (PD-L1^IC^ expression). PD-L1^IC^-positive tumors were observed in 129 (52.0%) of the TN tumors (Supplementary Table 1 and Supplementary Figure 3). Positive PD-L1 expression on tumor cells was significantly correlated with positive PD-L1^IC^ expression (Table 1).

**Table 1:** Patients and tumor characteristics in TNBC

	PD-L1-Positive		PD-L1-Negative		*P*
	*N* = 103 (41.5%)		*N* = 145 (58.5%)		
Age at diagnosis					
Mean (range)	57.4	(32–84)	61.8	(30–89)	**0.007**^a)^
Tumor size					
T1a/b (≤ 1 cm)	6	(5.8%)	14	(9.7%)	0.71^b)^
T1c (> 1 cm, ≤ 2 cm)	55	(53.4%)	71	(49.0%)	
T2 (> 2 cm, ≤ 5 cm)	39	(37.9%)	55	(37.9%)	
T3 (> 5 cm)	3	(2.9%)	5	(3.4%)	
Nodal status					
N0	67	(65.0%)	100	(69.0%)	0.84^b)^
N1 (1−3)	25	(24.3%)	33	(22.8%)	
N2 (4−9)	7	(6.8%)	7	(4.8%)	
N3 (≥10)	4	(3.9%)	4	(2.7%)	
Unknown			1	(0.7%)	
Pathological stage					
I	43	(41.7%)	63	(43.4%)	0.71^b)^
II	49	(47.6%)	71	(49.0%)	
III	11	(10.7%)	11	(7.6%)	
Nuclear grade					
1+2	19	(18.4%)	54	(37.2%)	**0.0015**^b)^
3	80	(77.7%)	88	(60.7%)	
Unknown	4	(3.9%)	3	(2.1%)	
Ki-67					
≤ 30%	6	(5.8%)	42	(29.0%)	**< 0.0001**^b)^
> 30%	83	(80.6%)	84	(57.9%)	
Unknown	14	(13.6%)	19	(13.1%)	
PD-L1 on immune cells					
Negative	17	(16.5%)	102	(70.3%)	**< 0.0001**^b)^
Positive	86	(83.5%)	43	(29.7%)	
TILs					
Low	16	(15.5%)	114	(78.6%)	**< 0.0001**^b)^
High	87	(84.5%)	31	(21.4%)	

^a)^ Logistic regression, ^b)^ Pearson's χ^2^ test.

### Patient survival

**Figure 2:**
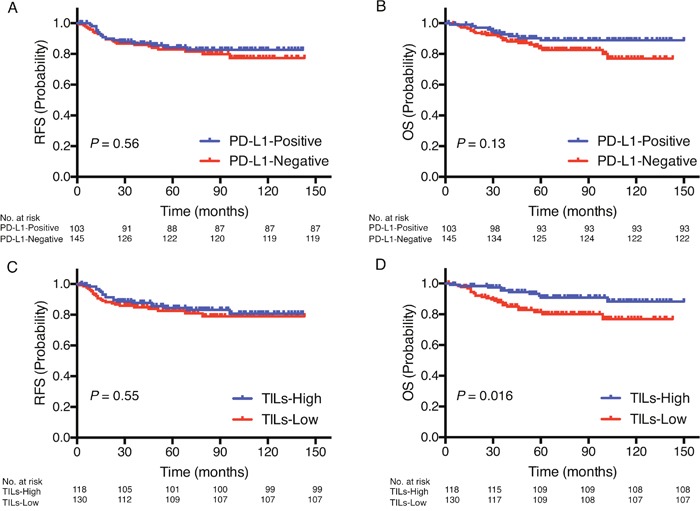
Prognostic value of PD-L1 expression and TILs status Kaplan-Meier curves showing estimated RFS **A**. and OS **B**. for PD-L1 expression as well as RFS **C**. and OS **D**. for TILs status. *P* values are for comparison of two groups.

The median follow-up in this cohort was 68 months (range 2–150 months). There was no significant difference in recurrence-free survival (RFS) and overall survival (OS) between patients with PD-L1-positive tumors and those with PD-L1-negative tumors (Figure 2A, 2B), and also there was no difference between patients with PD-L1^IC^-positive tumors and those with PD-L1^IC^-negative tumors (Supplementary Figure 4). Although there was no significant difference in RFS between patients with TILs-high and TILs-low tumors (Figure 2C), patients with TILs-high tumors had significantly better OS than those with TILs-low tumors (*P* = 0.016, Figure 2D).

**Figure 3:**
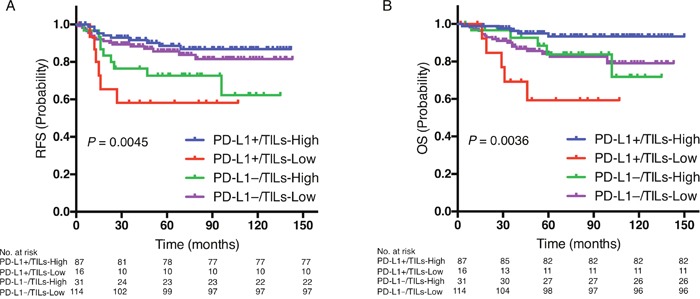
Prognostic value of the combination of PD-L1 expression and TILs status Kaplan-Meier curves showing estimated RFS **A**. and OS **B**. for PD-L1-positive/TILs-high, PD-L1-positive/TILs-low, PD-L1-negative/TILs-high, and PD-L1-negative/TILs-low. *P* values are for comparison of four groups.

The Cox proportional hazards model showed a significant interaction between PD-L1 and TILs (*P* = 0.0018 for RFS; *P* = 0.015 for OS, Table 2); that is, PD-L1 expression and TILs were not independent prognostic factors. The patients were therefore divided into four subgroups: PD-L1-positive/TILs-high, PD-L1-positive/TILs-low, PD-L1-negative/TILs-high and PD-L1-negative/TILs-low. Kaplan-Meier graphical analysis demonstrated that both RFS (*P* = 0.0045, Figure 3A) and OS (*P* = 0.0036, Figure 3B) differed significantly among the four subgroups. The treatment background of these four subgroups did not significantly differ (Supplementary Table 3).

**Table 2:** Interaction between PD-L1 and TILs in a Cox proportional hazards model

		Recurrence-free survival		Overall survival	
		Likelihood ratio χ^2^	*P*	Likelihood ratio χ^2^	*P*
PD-L1	(Positive vs. Negative)	0.07	0.79	0.04	0.85
TILs	(High vs. Low)	1.71	0.19	7.03	**0.008**
PD-L1*TILs		9.72	**0.0018**	6.00	**0.015**

*Interaction.

### Univariate and multivariate survival analysis

Univariate analysis of the clinicopathological characteristics revealed that tumor size (> 2 cm) and lymph node involvement were significantly related to poorer RFS and OS, while TILs-high tumors were significantly related to better OS (Table 3A). In addition, when we compared the PD-L1-positive/TILs-high subgroup, which had the longest RFS and OS, with other three subgroups, the PD-L1-positive/TILs-low subgroup had significant greater recurrence and death risks (for RFS: hazard ratio [HR] = 4.7, 95% confidence interval [CI] 1.6–12.7, *P* = 0.0067; for OS: HR = 8.4, 95% CI 2.3–30.3, *P* = 0.019, Table 3A).

Table 3

**Table:** A. Univariate analysis

Variables		Recurrence-free survival			Overall survival		
		HR	95% CI	*P*	HR	95% CI	*P*
Age	(> 50 vs. ≤ 50)	1.1	0.5−2.3	0.85	1.2	0.6−3.1	0.62
Tumor size	(> 2 cm vs. ≤ 2 cm)	2.7	1.5−5.1	**0.0015**	2.6	1.3−5.3	**0.0075**
Nodal status	(Positive vs. Negative)	2.8	1.5−5.1	**0.0011**	2.1	1.1−4.3	**0.032**
Nuclear grade	(3 vs. 1 and 2)	1.0	0.5−2.1	0.99	0.7	0.4−1.6	0.44
Ki-67	(> 30% vs. ≤ 30%)	1.8	0.8−5.2	0.21	1.2	0.5−3.2	0.72
PD-L1	(Positive vs. Negative)	0.8	0.4−1.5	0.56	0.6	0.3−1.2	0.13
PD-L1^IC^	(Positive vs. Negative)	0.6	0.3−1.1	0.09	0.7	0.4-1.4	0.35
TILs	(High vs. Low)	0.8	0.4−1.5	0.55	0.4	0.2−0.8	**0.015**
PD-L1*TILs	(PD-L1+/TILs-Low vs. PD-L1+/TILs-High)	4.7	1.6−12.7	**0.0067**	8.4	2.3−30.3	**0.0019**
	(PD-L1–/TILs-High vs. PD-L1+/TILs-High)	2.8	1.1−6.9	**0.031**	3.1	0.9−11.1	0.083
	(PD-L1–/TILs-Low vs. PD-L1+/TILs-High)	1.4	0.7−3.2	0.38	3.2	1.3−9.7	**0.013**

**Table:** B. Multivariate analysis

Variables		Recurrence-free survival			Overall survival		
		HR	95% CI	*P*	HR	95% CI	*P*
Tumor size	(> 2 cm vs. ≤ 2 cm)	2.4	1.3−4.5	**0.007**	2.1	1.1−4.5	**0.034**
Nodal status	(Positive vs. Negative)	2.3	1.2−4.2	**0.011**	1.9	0.9−3.8	0.083
PD-L1*TILs	(PD-L1+/TILs-Low vs. PD-L1+/TILs-High)	4.1	1.4−11.1	**0.014**	7.2	2.0−26.2	**0.0038**
	(PD-L1–/TILs-High vs. PD-L1+/TILs-High)	2.6	1.0−6.5	**0.043**	2.9	0.8−10.4	0.11
	(PD-L1–/TILs-Low vs. PD-L1+/TILs-High)	1.5	0.7−3.3	0.34	3.2	1.3−9.9	**0.011**

* Interaction; HR, hazard ratio; CI, confidence interval; +, positive; –, negative.

As individual factors, PD-L1 and TILs status were excluded from the multivariate analysis, because they were included in the four subgroups defined by combining the PD-L1 and TILs status. Age at diagnosis, nuclear grade, Ki-67 index and PD-L1^IC^ were also excluded from the multivariate analysis through the back elimination method. The multivariate analysis revealed that a tumor size (> 2 cm) and the PD-L1-positive/TILs-low subgroup were independent and negative prognostic factors for both RFS and OS (Table 3B).

## DISCUSSION

PD-L1 positivity in TNBC ranged from 19% to 58% in two previous studies [14, 19]. Differences in the cut-off value and primary antibody are likely reasons for the discrepancy in the percentages of PD-L1 expression between the two studies. Although studies have been conducted to analyze the relationship between PD-L1 and breast cancer, including all subtypes, the prognosis of patients with PD-L1-positive tumors remains controversial: PD-L1 was related to a poor prognosis [19–21], whereas PD-L1 expression was a good prognostic factor for breast cancer [16, 22], especially for basal-like tumors [17]. These controversial results might reflect the presence of multiple breast cancer subtypes, biological heterogeneity, or non-uniform methods for assessing PD-L1 status.

The International TILs Working Group recently issued recommendations for improving the consistency in scoring TILs, including detailed guidelines for annotating the prevalence of lymphocyte infiltration that may improve inter-observer reproducibility [23, 24]. We evaluated TILs according to these guidelines, and our data showed that patients with TILs-high tumors had significantly better OS than those with TILs-low tumors. This finding was consistent with the previous results of Pruneri et al. [8], who showed that each 10% increase in TILs strongly predicted better survival.

In the present study, PD-L1 expression was significantly correlated with higher levels of TILs. There few reports on the relationship between PD-L1 and TILs, and their results are controversial: higher CD8+ lymphocyte infiltration was related to lower PD-L1 expression in early-stage breast cancer [25], whereas PD-L1 expression showed a positive correlation with levels of infiltrating intratumoral CD8+ and FOXP3+ lymphocytes in breast cancer [18].

Our univariate analysis showed that PD-L1 expression on tumor cells was not a prognostic factor for RFS or OS. In combination, however, PD-L1 and TILs had a pronounced influence on patient prognosis, owing to interaction between PD-L1 and TILs. In addition, our multivariate analysis showed that the PD-L1-positive/TILs-low subgroup had the poorest prognosis, while the PD-L1-positive/TILs-high subgroup had the best prognosis among the four subgroups. Modulation of PD-L1 levels occurs via two major pathways, the intracellular (innate) signaling pathway mediated by PI3K/AKT/mTOR activation and/or the extracellular induced (adaptive) pathway mediated by IFNγ production by TILs and subsequent IFNGRs/JAK/STAT signaling in tumor cells [26]. When TILs levels are decreased in PD-L1-positive tumors, there may be aberrant activation of PI3K/AKT/mTOR signaling. Conversely, when levels of TILs are increased in PD-L1-positive tumors, there may be activation of IFNGRs/JAK/STAT signaling mediated by IFNγ production by TILs. Webb et al. showed that the PD-L1-positive/CD8+ tumors are associated with a better prognosis than PD-L1-negative/CD8+ or PD-L1-negative/CD8– tumors in high-grade serous ovarian cancer [27]. Teng et al. reported that four types of tumor microenvironment exist on the basis of their PD-L1 status and presence or absence of TILs [28]. Microenvirionments that are PD-L1-positive with TILs driving adaptive immune resistance are associated with the best prognosis.

This is the first report of an interaction between PD-L1 and TILs in breast cancer. The combination of PD-L1 and TILs may be the most robust factor predictive of prognosis in TNBC. Although PD-L1 expression is generally considered to indicate a poor prognosis [11–13], the prognosis of patients with PD-L1-positive/TILs-high tumors was improved. We therefore expect a positive effect of novel anti-PD-1/PD-L1 monoclonal antibody therapies in patients with PD-L1-positive/TILs-low tumors, for whom prognosis is currently poor. In fact, in a phase Ib trial for PD-L1-positive TNBCs (KEYNOTE-012), it was unclear whether PD-L1 expression was predictive of a clinical benefit with the PD-1 antibody pembrolizumab [29].

This study had several limitations. First, it included only retrospectively collected cases. Second, although we assessed the interactive effect PD-L1 and TILs, the causal relationship is not clear. Finally, these factors were not predictive of the response to treatments, including anthracycline- or taxane-based regimens (data not shown).

In conclusion, we report that PD-L1 expression on tumor cells is related to high TILs levels, and the combination of PD-L1-positive and TILs-low is associated with a poor prognosis in TNBC. Although additional research into the underlying mechanisms is necessary, these biomarkers may be useful for stratification for TNBC patients and for predicting their prognosis. Our findings support a rationale for the development of novel immune-targeted therapies, such as PD-1/PD-L1 inhibitors, for patients with TNBC.

## MATERIALS AND METHODS

### Patients

This study included 248 patients with primary TNBC who underwent resection without neoadjuvant chemotherapy at Kyushu University Hospital (Fukuoka, Japan), Hamanomachi Hospital (Fukuoka, Japan) or Kumamoto City Hospital (Kumamoto, Japan) between January 2004 and December 2014. About 20% of patients diagnosed with TNBC received neoadjuvant chemotherapy in our institutions and were excluded from this study. The patients received adjuvant treatment according to the National Comprehensive Cancer Network Guidelines for treatment of breast cancer (http://www.nccn.org/professionals/physician_gls/f_guidelines.asp#breast), the Clinical Practice Guideline of Breast Cancer by the Japanese Breast Cancer Society (http://jbcs.xsrv.jp/guidline/, in Japanese), and the recommendations of the St. Gallen International Breast Cancer Conference [30–33]. The treatment characteristics for the patients are shown in Supplementary Table 3. The study conformed to the principles of the Declaration of Helsinki and was approved by the Institutional Review Board of Kyushu University Hospital (No. 27-102).

### Immunohistochemistry (IHC)

Tumor subtypes were identified using IHC on surgically resected tissue. All resected specimens used for IHC were fixed (fixation was begun within 1 h) in 10% neutral buffered formalin for 6 to 72 h. ER-positive or PR-positive tissues were defined as ≥ 1% of tumor cells staining positive for ER or PR. Cancer specimens were defined as HER2-positive when HER2 IHC staining was scored as 3+ according to the standard criteria [34, 35], or when HER2 gene amplification was detected using fluorescence spectroscopy with in situ hybridization. The primary anti-PD-L1 antibody (monoclonal rabbit, E1L3N; Cell Signaling Technology, Beverly, MA) was used with a Ventana Discovery XT automated stainer (Ventana Medical Systems, Tucson, AZ) according to the manufacturer's protocol and using proprietary reagents. Briefly, slides were deparaffinized on the automated system with EZ Prep solution. A heat-induced antigen retrieval method was used in standard Cell Conditioning 1 with an incubation temperature of 95^°C^. The primary antibody was used at a 1:200 dilution and was incubated for 32 min. The secondary antibody was SignalStain Boost IHC Detection Reagent (Cell Signaling Technology). Slides were counterstained with hematoxylin, and a bluing reagent was used for counterstaining. Using the clinical trial assay to identify levels of PD-L1 expression on tumor cells that maximally predict clinical response to pembrolizumab [29], PD-L1 weak-positive was defined as membranous PD-L1 expression in 1–49% of tumor cells, and PD-L1 strong-positive was defined as expression in ≥ 50% of tumor cell (Supplementary Table 1 and Supplementary Figure 1). In addition, PD-L1^IC^-positive was defined as expression in ≥ 5% of tumor-infiltrating immune cells [12] (Supplementary Figure 3).

### Evaluation of tumor-infiltrating lymphocytes

TILs were assessed in hematoxylin and eosin-stained sections, carefully following the guidelines published by the International TILs Working Group to standardize TILs evaluation [23, 24] while blinded to the clinical information. These recommendations mainly propose a focus on stromal TILs. Cases were defined as TILs-high for ≥ 50% stromal TILs, which is also known as lymphocyte-predominant breast cancer, and as TILs-low for < 50% stromal TILs (Supplementary Figure 2).

### Statistics

Logistic regression was used to compare continuous variables and χ^2^ tests were used to compare categorical variables between the PD-L1-positive and PD-L1-negative groups. The survival endpoints evaluated were RFS and OS. RFS was defined as the time from surgery to recurrence, including both local relapse and metastatic disease. OS was defined as the time from surgery until the date of death from any cause. Survival curves were generated using the Kaplan-Meier method and compared with the log-rank test. Hazard ratios were calculated using Cox proportional hazards regression. Values of *P* < 0.05 were considered statistically significant. Statistical analysis was carried out using JMP^®^ 11 (SAS Institute Inc., Cary, NC).

## SUPPLEMENTARY MATERIALS FIGURES AND TABLES


