# Supplementary material for: The combination of PD-L1 expression and decreased tumor-infiltrating lymphocytes is associated with a poor prognosis in triple-negative breast cancer
Source: Oncotarget. 2017 Jan 17;8(9):15584–92. doi: 10.18632/oncotarget.14698 (PMC5362507; doi:10.18632/oncotarget.14698)
Supplement: Supplementary file 1 [file oncotarget-08-15584-s001.pdf]

The combination of PD-L1 expression and decreased tumor-infiltrating lymphocytes is associated with a poor prognosis in triple-negative breast cancer

SUPPLEMENTARY FIGURES AND TABLES

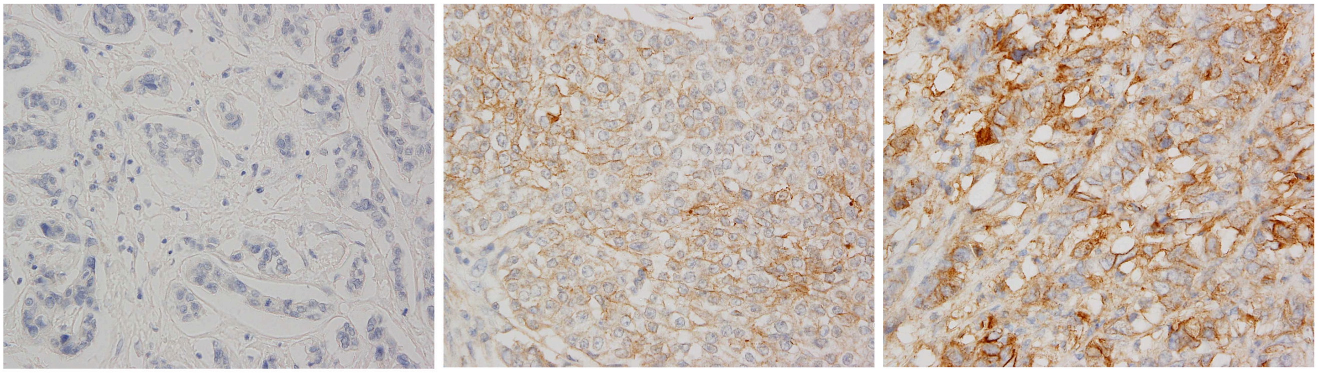

Negative Weak-positive Strong-positive

Supplementary Figure 1: Immunostaining of PD-L1 on tumor cells (×400).

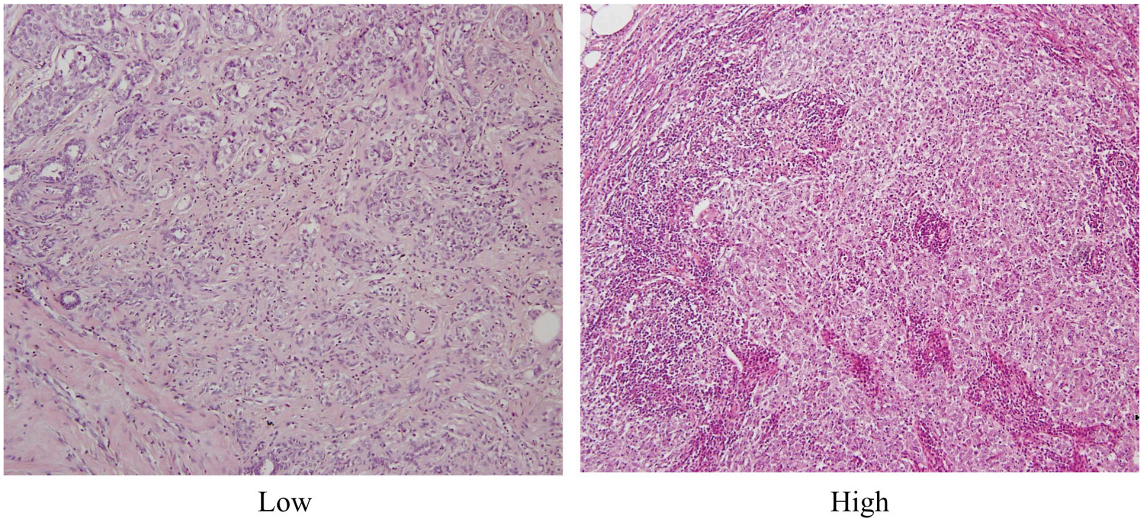

Supplementary Figure 2: Evaluation of TILs using hematoxylin and eosin staining (×100).

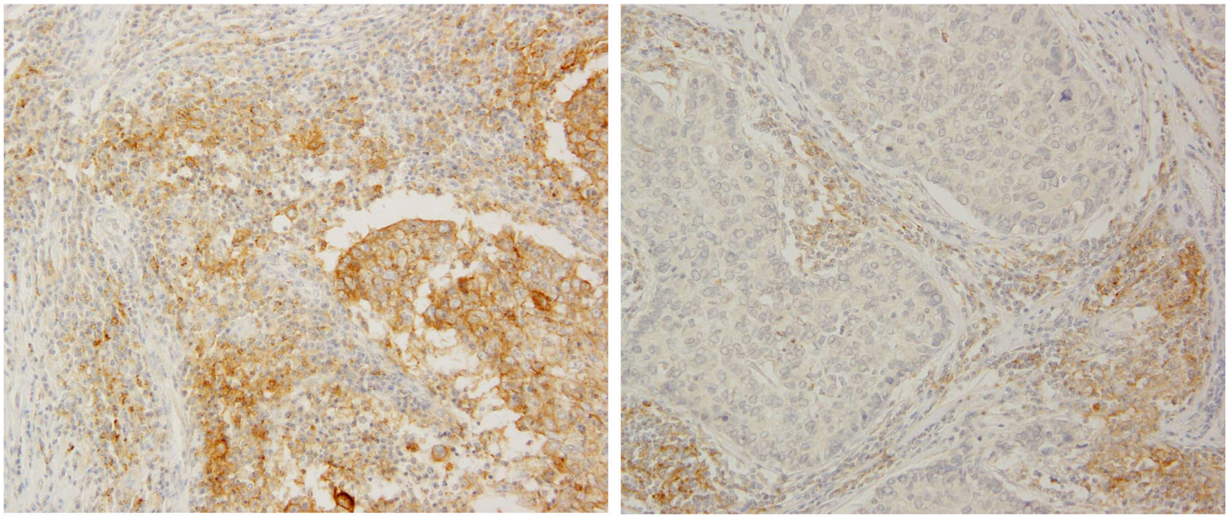

Immune cells: IHC 3
(Tumor cells: Strong positive)

Immune cells: IHC 3
(Tumor cells: Negative)

Supplementary Figure 3: Immunostaining of PD-L1 on immune cells (×200).

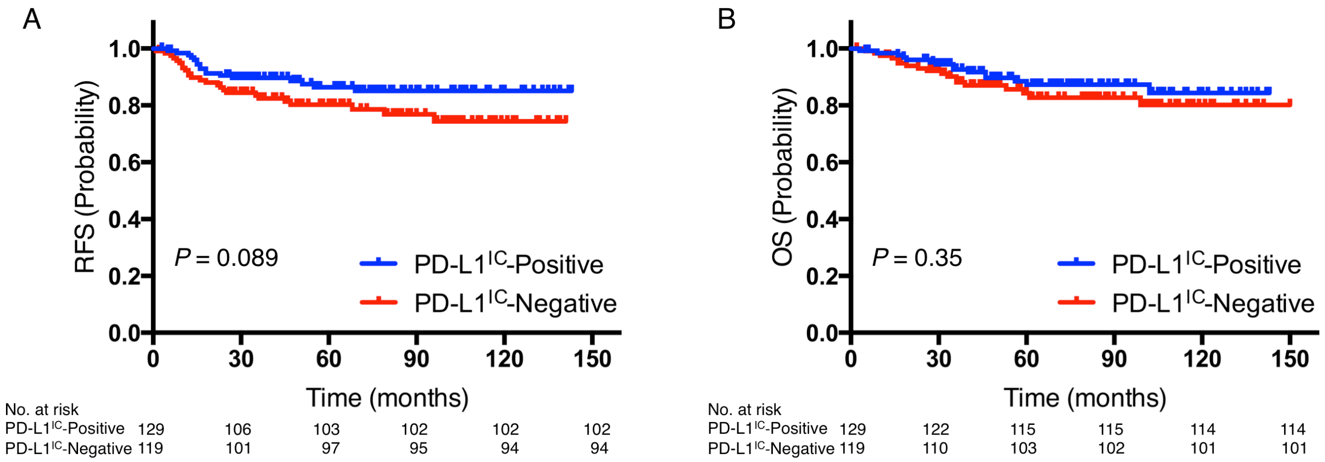

Supplementary Figure 4: Prognostic value of PD-L1^{IC} expression. Kaplan-Meier curves showing estimated RFS **A.** and OS **B.** for PD-L1^{IC} expression. *P* values are for comparison of two groups.

Supplementary Table 1: Assessment of PD-L1 expression on tumor and immune cells

	Negative		Positive	
Tumor cells			Weak	Strong
Positive staining rate	< 1%		1−49%	≥ 50%
Number of cases	145		65	38
Immune cells	IHC 0	IHC 1	IHC 2	IHC 3
Positive staining rate	< 1%	≥ 1% and < 5%	≥ 5% and < 10%	≥ 10%
Number of cases	91	28	43	86

Supplementary Table 2: Patient and tumor characteristics based on TILs levels

	TILs-High		TILs-Low		*P*
	N = 118 (47.6%)		*N* = 130 (52.4%)		
Age at diagnosis					
Mean (range)	56.5	(32–84)	63.2	(30–89)	**< 0.0001**[a)]
Tumor size					
T1a/b (≤ 1 cm)	8	(6.8%)	12	(9.2%)	0.70[b)]
T1c (> 1 cm, ≤ 2 cm)	64	(54.2%)	62	(47.7%)	
T2 (> 2 cm, ≤ 5 cm)	43	(36.5%)	51	(39.2%)	
T3 (> 5 cm)	3	(2.5%)	5	(3.9%)	
Nodal status					
N0	75	(63.6%)	92	(70.8%)	0.45[b)]
N1 (1−3)	32	(27.1%)	26	(20.0%)	
N2 (4−9)	6	(5.1%)	8	(6.1%)	
N3 (≥ 10)	5	(4.2%)	3	(2.3%)	
Unknown			1	(0.8%)	
Pathological stage					
I	50	(42.4%)	56	(43.1%)	0.52[b)]
II	55	(46.6%)	65	(50.0%)	
III	13	(11.0%)	9	(6.9%)	
Nuclear grade					
1+2	21	(17.8%)	52	(40.0%)	**0.0003**[b)]
3	91	(77.1%)	77	(59.2%)	
Unknown	6	(5.1%)	1	(0.8%)	
Ki-67					
≤ 30%	6	(5.1%)	42	(32.3%)	**< 0.0001**[b)]
> 30%	95	(80.5%)	72	(55.4%)	
Unknown	17	(14.4%)	16	(12.3%)	
PD-L1 on tumor cells					
Negative	31	(26.3%)	114	(87.7%)	**< 0.0001**[b)]
Positive	87	(73.7%)	16	(12.3%)	
PD-L1 on immune cells					
Negative	27	(22.9%)	92	(70.8%)	**< 0.0001**[b)]
Positive	91	(77.1%)	38	(29.2%)	

[a)] Logistic regression, [b)] Pearson's χ^2 test.

Supplementary Table 3. Treatment characteristics for patients with TNBC

	PD-L1-Positive				PD-L1-Negaive				***P***
	TILs-High		TILs-Low		TILs-High		TILs-Low		
Surgical treatment									
Breast-conserving surgery	58	(66.7%)	8	(50.0%)	19	(61.3%)	63	(55.3%)	0.35
Mastectomy	29	(33.3%)	8	(50.0%)	12	(38.7%)	51	(44.7%)	
Adjuvant chemotherapy									
Anthracycline-based regimens									
AC, EC, FEC	37	(42.5%)	4	(25.0%)	12	(38.7%)	36	(31.6%)	0.09
EC+PTX, FEC+DTX	29	(33.3%)	7	(43.8%)	10	(32.3%)	19	(16.7%)	
Non-anthracycline-based regimens									
TC, DTX	2	(2.3%)	0		0		7	(6.1%)	
CMF	1	(1.2%)	1	(6.3%)	0		3	(2.6%)	
Others	1	(1.2%)	0		1	(3.2%)	2	(1.7%)	
No treatment	17	(19.5%)	4	(25.0%)	8	(25.8%)	46	(40.4%)	
Unknouwn	0		0		0		1	(0.9%)	
Duration of adjuvant chemotherapy									
Mean (range)	5.4	(3-9)	6.7	(6-8)	4.5	(3-6)	4.6	(1.5-8)	0.16

AC, doxorubicin (60mg/m^2) and cyclophosphamide (600mg/m^2) every 3 weeks; EC, epirubicin (90mg/m^2) and cyclophosphamide (600mg/m^2) every 3 weeks; FEC, 5-fluorouracil (500mg/m^2), epirubicin (100mg/m^2), and cyclophosphamide (500mg/m^2) every 3 weeks; TC, docetaxel (75mg/m^2) and cyclophosphamide (600mg/m^2) every 3 weeks; DTX, docetaxel (75mg/m^2) every 3 weeks; CMF, cyclophosphamide (100mg/m^2), methotrexate (40mg/m^2), and 5-fluorouracil (600mg/m^2) every 4 weeks.
